# Detection of the emerging tick-borne pathogen, Tamdy virus, in *Dermacentor marginatus* ticks, southeastern Türkiye

**DOI:** 10.1590/S1984-29612026009

**Published:** 2026-05-25

**Authors:** Ender Dincer, Mehmet Ozkan Timurkan

**Affiliations:** 1 Dokuz Eylul University, Faculty of Veterinary Medicine, Department of Virology, Izmir, Turkey; 2 Atatürk University, Faculty of Veterinary Medicine, Department of Virology, Erzurum, Turkey

**Keywords:** Tick- borne pathogens, tick, Tamdy virus, Türkiye, Patógenos transmitidos por carrapatos, carrapato, vírus Tamdy, Turquia

## Abstract

Tamdy virus (TAMV) is an emerging tick-borne virus in the family Nairoviridae, identified as a human pathogen causing febrile illness after tick exposure. This study aimed to detect and characterise TAMV in ticks collected from non-endemic regions of Türkiye. A total of 529 ticks were sampled from six provinces and identified as *Haemaphysalis parva* (27%), *Rhipicephalus bursa* (26%), *Haemaphysalis punctata* (20.7%), *Dermacentor marginatus* (17.2%), and *Ixodes ricinus* (8.8%). Screening with a single-step generic PCR revealed one TAMV-positive pool out of 96. For characterisation, the S segment of TAMV was amplified using two primer sets. Phylogenetic analyses of the partial L gene and full S segment showed that the sequence from Osmaniye sample clustered closely with the TT1 isolate from Konya, Türkiye. Amino acid comparison indicated that Turkish isolates were more similar to each other than to those from Russia and China, forming a distinct clade within TAMV. Minor amino acid differences between the Turkish isolates suggest that TAMV diversity in Türkiye is shaped by internal evolution rather than external introductions. These findings highlight the importance of continuous surveillance of tick-borne zoonotic viruses and suggest that TAMV may have a wider vector range and geographic distribution than previously recognised.

## Resumo

O vírus Tamdy (TAMV) é um vírus emergente transmitido por carrapatos da família Nairoviridae, identificado como um patógeno humano que causa doença febril após exposição a carrapatos. Este estudo teve como objetivo detectar e caracterizar o TAMV em carrapatos coletados em regiões não endêmicas da Turquia. Um total de 529 carrapatos foram amostrados de seis províncias e identificados como *Haemaphysalis parva* (27%), *Rhipicephalus bursa* (26%), *Haemaphysalis punctata* (20,7%), *Dermacentor marginatus* (17,2%) e *Ixodes ricinus* (8,8%). A triagem com PCR genérico de etapa única revelou um *pool* positivo para TAMV em 96. Para caracterização, o segmento S do TAMV foi amplificado usando dois conjuntos de primers. As análises filogenéticas do gene L parcial e do segmento S completo mostraram que a sequência da amostra de Osmaniye se agrupou estreitamente com o isolado TT1 de Konya, Turquia. A comparação de aminoácidos indicou que os isolados turcos eram mais semelhantes entre si do que os da Rússia e da China, formando um clado distinto dentro do TAMV. Pequenas diferenças de aminoácidos entre os isolados turcos sugerem que a diversidade do TAMV na Turquia é moldada pela evolução interna, e não por introduções externas. Essas descobertas destacam a importância da vigilância contínua de vírus zoonóticos transmitidos por carrapatos e sugerem que o TAMV pode ter um alcance vetorial e uma distribuição geográfica mais amplos do que se reconhecia anteriormente.

## Introduction

Ticks are involved in the maintenance and transmission of more than 38 identified virus species. Their distinctive biological characteristics enhance their capacity to disseminate these pathogens to susceptible hosts (Parola & Raoult, 2001; Dantas-Torres et al., 2012; Shah et al., 2023). Current evidence indicates a global rise in the incidence of tick-borne infections, likely driven by environmental and demographic changes over recent decades. As a result, these infections are now considered credible risks of emergence (Rich et al., 2023; Vayssier-Taussat et al., 2015). Therefore, tick surveillance serves as an essential tool for understanding the spatio-temporal dynamics of virus activity and for predicting potential outbreaks.

Tamdy virus (TAMV, new name *Orthonairovirus tomdiense*) is a tick-borne RNA virus with a single-stranded positive-sense genome that is 15 kb long, belonging to the *Bunyavirales* order and the *Orthonairovirus* genus in the *Nairoviridae* family. The genome has three parts: the large (L) part makes RNA-dependent RNA polymerase, the medium (M) part makes envelope glycoproteins (Gc and Gn), and the small (S) part makes the nucleocapsid protein (NP). The *Nairoviridae* family includes various viruses that cause disease in humans and animals. The best known of these, Crimean-Congo haemorrhagic fever virus (CCHFV), has spread over a wide geography from Africa to Southern Europe, from the Far East to the Arabian Peninsula (Spengler et al., 2019). The other members of these groups, such as Tofla virus (TFLV, *Orthonairovirus japonicum*) (Shimada et al., 2016), Dugbe virus (*Orthonairovirus dugbeense*) (David-West, 1973), Erve virus *(Orthonairovirus erveense)* (Chastel et al., 1989), Issyk-Kul virus (*Orthonairovirus issykkulense*) (Brinkmann et al., 2020), and Kasokero virus *(Orthonairovirus kasokeroense)* (Schuh et al., 2020), have been listed as well-known and prevalent tick-borne human viral pathogens. The mechanisms by which these viruses cause disease remain poorly understood, and no effective antiviral treatments or vaccines are currently available. Consequently, emerging tick-borne viruses (TBVs) represent an increasing threat to public health by complicating efforts for disease prevention and control. Recent advances in metagenomic sequencing have facilitated the discovery of numerous novel tick-associated nairoviruses, including Yezo virus (Kodama et al., 2021), Songling virus (Ma et al., 2021), and Tacheng tick virus-1 (TcTV-1) (Liu et al., 2020), leading to human symptomatic infections generally characterised by febrile illnesses. TAMV was initially reported in Asiaticum ticks in the Tamdinsky district of the Bukhara region, Uzbekistan, in 1971 (L’vov et al., 1976). Further studies demonstrated the presence of TAMV in various tick species in Central Asian countries, such as Uzbekistan, Turkmenistan, Kyrgyzstan, Kazakhstan, and Armenia. More importantly, infection of TAMV has been detected in patients with febrile illness in Kyrgyzstan and Kazakhstan (L’vov et al., 1984). In 2019, TAMV was first found in *Hyalomma asiaticum* ticks taken from 2-humped Bactrian camels in Xinjiang, China (Zhou et al., 2019)*.* In 2021, in addition to the detection of the TAMV in *Hyalomma asiaticum* ticks, the first serological evidence of human TAMV infection was also carried out in the same region of China (Moming et al., 2021). The detection of TAMV in *Hyalomma dromedarii* ticks collected from the Arabian Peninsula showed that the virus had spread to a wider area than originally recognised (Zakham et al., 2021). Finally, another study revealed the presence of virus-specific antibodies in the blood of domestic and wild animals as well as various tick species in China (Moming et al., 2024).

The nucleoprotein (NP) of TAMV is responsible for encapsidation of the viral genome in the life cycle of the virus. In addition, it has some functional properties, including promoting genomic replication and inhibiting apoptosis. Especially, recent studies on NP of CCHFV have gained momentum. In these studies, the protective effects of antibodies against CCHFV NP have been described. Firstly, it has also been shown that passive transfer of NP-immun serum provides protection against fatal CCHFV infections. Secondly, the protective efficacy observed following the passive transfer of immune sera suggests that anti-NP antibodies could serve as viable therapeutics for CCHFV. Ongoing studies are currently evaluating this approach (Ergünay et al., 2020; Leventhal et al., 2024). The determination of the functional properties of the NP of the TAMV, which is in the same family as the CCHFV, can provide its use in various areas. In particular, the effects of the amino acid diversity of the S segment of the TAMV on the function of the NP can be determined, and active structures can be revealed. Finally, this may contribute to treatment options against TAMV.

The prevalence of TAMV in Türkiye was lacking until 2018, when TAMV was detected in a tick, *Hyalomma marginatum*, collected from a gerbil (*Meriones tristrami*) in Sultan Sazlığı Wildlife Park, Kayseri, Central Anatolia Region (Brinkmann et al., 2018). Subsequently, TAMV was reported in *Hyalomma aegyptium* ticks collected from Konya, Central Anatolia Region (Ergünay et al., 2020). To date, there is no data or publication about TAMV other than these two field surveys in Türkiye. In Türkiye, located at the intersection of Europe, Asia, and Africa, has diverse climatic zones. These, along with bird migration routes, have contributed to the distribution of various tick species and TBVs that infect humans and animals (Bursali et al., 2012). The newly identified TBVs, such as Jingmen tick virus (JMTV) (Dinçer et al., 2019), Meram virus, TAMV (Ergünay et al., 2020) and TcTV-1 (Dincer et al., 2023) appears to be overshadowed by studies focusing on CCHFV in Türkiye. Therefore, the potential tick vectors, reservoir hosts, and geographic distributions of these viruses are still poorly understood. To address this knowledge gap, we performed molecular screening and full S segment analyses for TAMV in ticks collected from livestock (sheep and goats) across several provinces in Türkiye.

## Material and Methods

### Tick collection and processing

Ticks were collected from Osmaniye and Hatay provinces in the Mediterranean region; Malatya, Erzincan, and Bingöl in the Eastern Anatolia region; and Amasya in the Black Sea region between March and August 2025. Sampling was conducted monthly throughout the study period, and the number of collected specimens, classified by tick species and province, is summarized in Table 1. Ticks were collected directly from the body surface of goats and sheep during routine physical examinations using fine-tipped forceps. No questing ticks were included in this study. The collected specimens were placed into labeled tubes and transported to the laboratory under cold chain conditions for further processing. Subsequently, morphological identification of ticks was carried out using taxonomic keys (Estrada-Pena et al., 2017; Walker, 2000). To eliminate surface contaminants, each tick was washed twice with sterile phosphate-buffered saline (PBS). Tick pools comprising 1 to 10 individuals were then formed according to species, sex, developmental stage (adult or nymph), collection site, and host animal. Only ticks sharing the same characteristics for these criteria were combined within the same pool. Each pool was subsequently processed as a single sample for molecular analysis. The grinding process was performed using tungsten carbide or stainless steel beads (Qiagen, Hilden, Germany) in 300–900 µL of Dulbecco’s phosphate-buffered saline, supplemented with 1% L-glutamine and 5% foetal bovine serum, and then the supernatant was collected with centrifugation at 6000 rpm at 4 °C for 10 min. These supernatants were then stored at –80°C until the extraction step. Viral nucleic acid was extracted using the High Pure Viral Nucleic Acid Kit (Roche Diagnostics, Germany) according to the manufacturer’s instructions. Complementary DNA (cDNA) was also synthesised using the Xpert cDNA Synthesis Kit (GRiSP Research Solutions, Portugal) according to the manufacturer’s instructions. Negative extraction and RT-PCR controls were used in every 24 samples to eliminate cross-contamination. Additionally, extraction and RT-PCR procedures were performed in different rooms in line with general laboratory rules. The setup in our study was firstly the detection of the L segment of the virus, and then the complete genomic characterization of the S segment in positive samples.

**Table 1:** Distrubution of the tick samples according to species and collection areas.

	**Blacksea region**		**Eastern Anatolia**						**Mediterranean**				**Total**
	**Amasya**		**Malatya**		**Erzincan**		**Bingöl**		**Hatay**		**Osmaniye**		
	♀	♂	♀	♂	♀	♂	♀	♂	♀	♂	♀	♂	
*Rhipicephalus bursa*	20	10	15	0	10	8	18	5	23	11	15	3	138
*Dermacentor marginatus*	5	0	9	3	8	7	16	10	6	5	12	10	91
*Haemaphysalis punctata*	19	5	11	7	12	5	23	0	6	2	13	7	110
*Haemaphysalis parva*	18	12	14	7	9	1	15	10	14	9	22	12	143
*Ixodes ricinus*	14	7	0	0	7	4	0	0	9	1	5	0	47
Total	76	34	49	17	46	25	72	25	58	28	67	32	529
	110(20.7%)		66 (12.4%)		71(13.4%)		97(18.3%)		86(16.2%)		99(18.7%)		
Pool	20(12.5%)		10(10.4%)		12(12.5%)		18(18.75%)		15(15.6%)		21(21.8%)		96

### Detection of TAMV in ticks

To detect the presence of TAMV infection in tick pools, a generic single-round PCR protocol for orthonairoviruses was performed using primers targeting the viral polymerase central motif A region on the L genomic segment (Honig et al., 2004). Motif A is a highly conserved domain within the RNA-dependent RNA polymerase (RdRp) of orthonairoviruses, which plays a crucial role in the catalytic activity of nucleotide incorporation during viral RNA synthesis. Due to its high sequence conservation across nairoviruses, this region serves as a reliable target for generic detection of Tamdy and related orthonairoviruses. In sum, PCR amplification was done with a few modifications: initial 5 min denaturation at 95 °C, followed by 40 cycles at 95 °C for 40 s, 42–50 °C (ramping over the 40 cycles) for 1 min, and 72 °C at 1 min, with a final extension at 72°C at 10 min respectively. The amplification products (product size, 489 base pairs) were run with 1% agarose gel electrophoresis, stained with SYBR safe DNA gel stain (Thermo Fisher Scientific, Germany), and visualised in a ChemiDoc XRS+ imaging system (BioRad, Germany). DNase/RNase-free water was used as a negative control for each PCR. For RT-PCR and viral nucleic acid extraction steps, biosafety level 2 class cabinets were used at various times and in various laboratories.

### Full genome amplification of the S segment of TAMV

The full genome of the S segment of TAMV was amplified in two seperates PCRs, targeting S gene region in lengths of 1,072 and 1,056 bp as described previously (Zhou et al., 2019). The S segment region was amplified using FS1-5’-CACACCCAAACTTTACACTTTAGG -3’ and RS1 - 5’- TCAACTTCCTTGGGCAATCCT-3’ for 1.072bp; FS2-5’-CCTTGTTCTAGCTCTCAATGACC-3’ and RS2-5’- CACACCCAAAGCAAATTGCTC- 3) for 1,056 bp, respectively.

### Cell culture and virus isolation

For TAMV isolation, cell lines, Vero cells, and Vero E6 available in the laboratory archives were preferred. The positive tick homogenate was inoculated in various rates (300-600 µL) onto cells in 25 cm^2^ flasks, taking into account previous similar laboratory experiences. After the adsorption period, growth medium was added to the cells, and they were observed every other day for possible pathologies. Three blind passages were performed, and then cells were harvested between 10 and 12 days, and inocula were investigated in RT-PCR for the presence of possible viruses.

### Sequencing and data analyses

The PCR products were purified using the PureLink PCR Purification Kit (Thermo Fisher Scientific, Hennigsdorf, Germany) and subsequently subjected to DNA sequencing using the BigDye Terminator v3.1 Cycle Sequencing Kit (Thermo Fisher Scientific) on an ABI PRISM 3500xL Dx Genetic Analyser (Thermo Fisher Scientific). Similarity searches were performed in the National Centre for Biotechnology Information (NCBI) databases using the BLASTn, MEGABLAST (BLASTn optimised for highly similar sequences), and BLASTp algorithms. Alignment and pairwise comparisons of nucleotide and deduced amino acid sequences were investigated using the CLUSTAL W program. Moreover, nucleotide similarity and bootscan plots were generated using SimPlot (version 3.5.1) (Lole et al., 1999). For phylogenetic analyses, results of DNA sequencing were evaluated using Maximum-likelihood method based on the Tamura 3-parameter model with 500 bootstrap replicates in MEGA X version (Kumar et al., 2018). In the phylogenetic analysis, reference strains from GenBank, including all genera of the *Nairoviridae* family possible to detect in ticks, and strains obtained from our study were used. For this purpose, two separate phylogenetic tree were created for both the complete S segment (nucleocapsid protein gene) and the partial L segment (RdRp gene).

## Results

### Tick collection and identification of TAMV

A total of 529 tick were collected from six provinces: Amasya from the Black Sea; Malatya, Erzincan, and Bingöl from East Anatolia; and Hatay and Osmaniye from the Mediterranean in Türkiye. Species identification demonstrated that these tick samples were listed in *Haemaphysalis parva* (n=143; 27%), *Rhipicephalus bursa* (n=138; 26%), *Haemaphysalis punctata* (n= 110; 20.7%), *Dermacentor marginatus* (n=91; 17.2%) and *Ixodes ricinus* (n= 47; 8.8%) (Table 1). The 96 pools containing 1 to 10 individuals were created according to the criteria previously stated. One tick pool (pool 44) tested positive for TAMV by single-round PCR. The minimum infection rate (MIR) was calculated as 1.04% (1 positive pool out of 96 tested). The female pool, Osmaniye 44(OS44), of *Dermocentor marginatus* obtained from Osmaniye province was positive for TAMV. Moreover, the positive tick were collected from a sheep flock in the rural area of ​​Osmaniye province in June.

Inocula prepared from the TAMV-positive pool were seeded into Vero and Vero E6 cell line. No CPE development was observed at the end of the three blind passages. Unfortunately, virus isolation could not be achieved for TAMV. No positivity was detected in the PCR test controls performed after three blind passages in cell cultures.

### Phylogenetic analyses of partial L and full S segments

**Figure 1:**
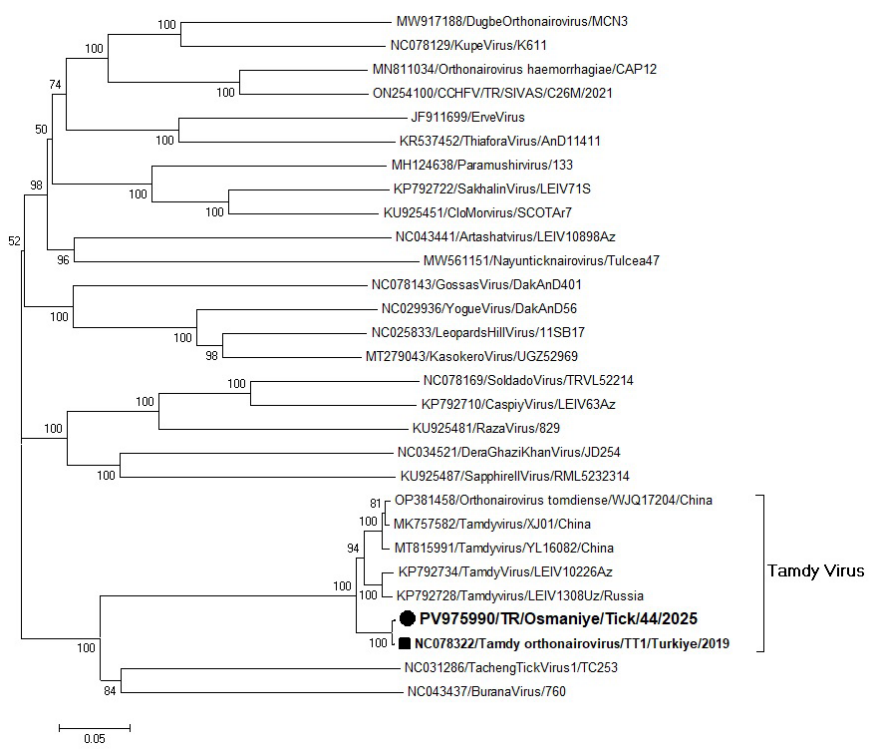
Maximum likelihood analysis was generated using partial L segment sequences (477 nucleotides) with specific members of the Nairoviridae family. The Tamdy virus sequence identified in the present study is represented in a bold circle and indicated with the GenBank accession number, province, host, and pool code. The black square symbol indicates the previously detected Tamdy virus sequence in Türkiye. Reference sequences from globally reported virus strains are denoted by their GenBank accession numbers, along with virus and strain/isolate names where applicable. The Uukuniemi virus (family Phenuiviridae) was included as an outgroup to root the phylogenetic tree.

**Figure 2:**
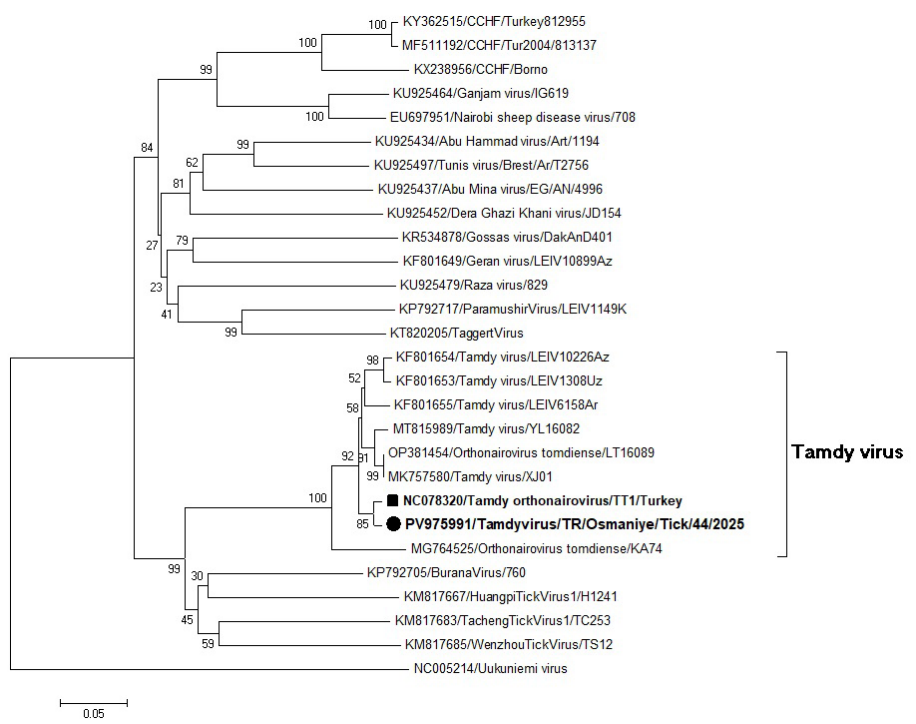
Maximum likelihood analysis was generated using the S segment (2002 nt) sequence with specific members of the Nairoviridae family. The Tamdy virus sequence identified in the present study is represented in a bold circle and indicated with the GenBank accession number, province, host, and pool code. The Tamdy virus sequence previously detected in Türkiye is indicated with a black square symbol. Reference sequences from globally reported virus strains are denoted by their GenBank accession numbers, along with virus and strain/isolate names where applicable. The Uukuniemi virus (family Phenuiviridae) was included as an outgroup to root the phylogenetic tree.

For the positive sample, we performed two separate PCRs for the S gene region. Subsequently, two PCR products were amplified as 1.072bp and 1.056bp, respectively. For simplot analyses of the S segment of TAMV, reference sequences were obtained from the geneBank. These sequences were listed as MK757582 and OP381458 from China, NC078322 from Türkiye, and KP792728 from Russia, respectively. The nucleocapsids in the Nairoviridae family, including the TAMV and TFLV/HAZV/Meram viruses, consist of 483 and 485 amino acids, respectively. We investigated particular structural and functional features of the TAMV nucleocapsid proteins for local and global TAMV virus sequences, including the previously detected TT1 isolate from Türkiye. The phylogenetic analyses (Figure 1) of the partial L segment showed that the TAMV sequence characterised in this study was closely related to the virus previously reported from Konya province in central Anatolia and created a separate group from the Russian and Chinese isolates. Moreover, sequence alignment analysis demonstrated that the nucleotide similarity rate between OS44 and TT1 isolate was 98%. The slightly lower similarity (94% - 96%) was found between global viruses and Turkish strains. Sequence comparison of the S segment indicates that OS44 was highly similar (99%) to the previously described TT1 isolate from Konya, central Anatolia. Moreover, the similarity rate of S segment in turkish strains and other global TAMV isolates was found as 94%- 95% and formed a separate group within Nairoviruses (Figure 2).

**Figure 3:**
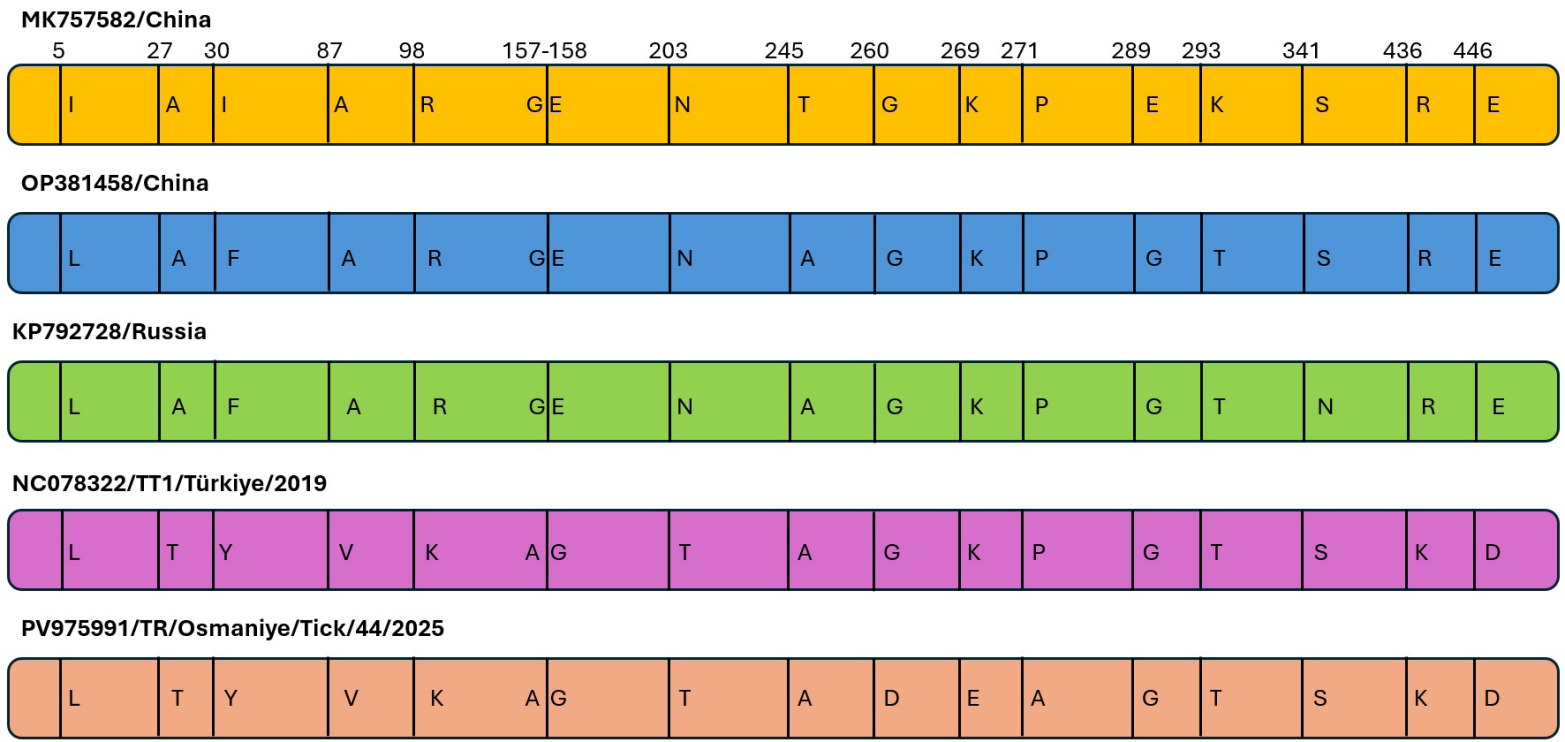
Schematic diagram of amino asit sequences of Tamdy virus nucleocapsid region reported in the present and previous studies.

In the present study, amino acid alignment based on S segment analyse showed that OS44 and TT1 isolate had high amino acid similarity (98%). However, some amino acid changes on NPs of OS44 and TT1 isolate were detected at residues 260 (D→G), 269 (K→E), and 271 (P→A). Such as Turkish and global isolates (Russia and China), more amino acid changes were observed at residues 87 (V→A), 98 (K→R), 157 (A→G), 158 (G→E), 203 (T→N), 436 (K→R), and 446 (D→E), respectively. Therefore, these findings suggest that the nucleocapsid structure of Turkish strains is more different than other isolates (Figure 3). In nairoviruses, NPs may nonspecifically associate with nairovirus RNA sequences to form a ribonucleoprotein (RNP) complex [32]. For example, two positively charged regions of CCHFV NP carry out RNA binding. The CCHFV NP is also associated with the antiviral defence factor Max and the apoptosis mediator caspase-3 (Ergünay et al., 2020). Especially, particular residues are necessary for successful in vitro replication. These were reported in all TAMVs with minor modifications (Andersson et al., 2004).

### Simplot analyse

**Figure 4:**
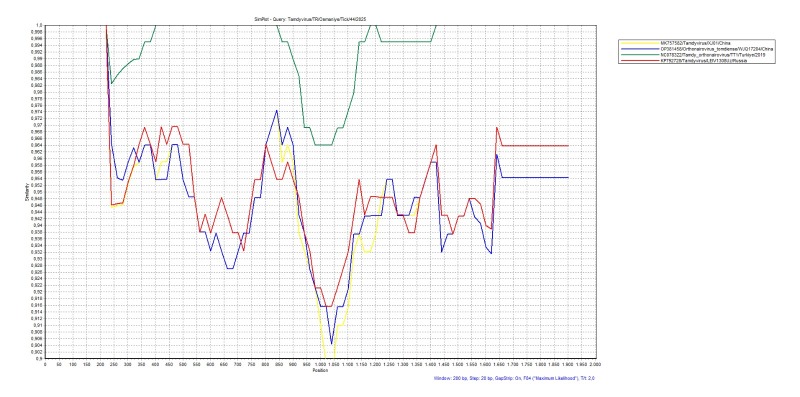
Similarity plots of Tamdy virus genome alignment (S segment) with members of the Tamdy virus species (GapStrip: On, Reps: 1000, Maximum Likelihood, T/t: 2.0). The curves indicate comparisons between the target and reference genomes (MK757582 / XJ01 / China, OP381458 / WJQ17204 / China, NC_078322 / TT12019 / Türkiye, KP792728 / LEIV1308Uz / Russia). Each point plotted indicates percent identity within a sliding window 200 bp wide centred on the position plotted, with a step size between points of 20 bp.

The SIMPLOT analysis showed high nucleotide similarity (99%) between OS44 and TT1 isolate. However, some variabilities were largely detected in 250–400, 850–1150, and 1200–1400 positions between OS44 and TT1 isolate. In addition, the OS44 had higher genetic variability than the Russia and China isolates. More importantly, the Simplot data suggested that all Turkish strains were more distant than global isolates (China and Russia) (Figure 4). Although the number of available TAMV sequences is limited, phylogenetic and similarity plot analyses of the S segment suggest that Turkish TAMV strains cluster closely together and exhibit certain genetic divergence from strains reported in other countries. However, due to the small dataset, it remains premature to conclude that genetic exchanges occur exclusively within Türkiye without interactions with global lineages. To better understand and elucidate this, all segments of TAMV isolates in Türkiye should be investigated and characterised using a variety of genetic analysis protocols.

## Discussion

During the past decade, several TBVs associated with febrile illnesses in humans have been identified in both ticks and domestic or wild animals in Türkiye. These findings highlight the considerable viral diversity carried by ticks infesting humans and animals. In this context, our study provides additional evidence for the circulation of TAMV in southeastern Türkiye, emphasizing the potential role of local tick populations in the ecology of emerging TBVs. Among the tick-borne nairoviruses reported in Türkiye, CCHFV, TcTV-1, and TAMV are of particular interest due to their zoonotic potential. While CCHFV has long been recognized as a major public health concern with substantial human morbidity and mortality, the epidemiology and clinical relevance of TcTV-1 and TAMV remain poorly understood. To date, there are no available data on human or livestock infections or seroprevalence related to TAMV in Osmaniye province. Nevertheless, the detection of TAMV in this region underscores the need for continued surveillance and further investigations to clarify its circulation dynamics and potential impact on public health. Since the TAMV was first documented in Uzbekistan and in other Central Asian countries in the 1970s, in the last 10 years, it has expanded both tick vectors and geographical distribution in the world.

This study describes TAMV prevalence in 539 ticks (comprising *R. bursa*, *D. marginatus*, *H. punctata*, *H. parva*, and *I. ricinus*) collected from domestic animals across six Turkish provinces (Osmaniye, Hatay, Malatya, Erzincan, Bingöl, and Amasya). In total, 96 tick pools were screened for TAMV using a single-round generic PCR protocol. In the present study, TAMV RNA was detected for the first time in *D. marginatus*, suggesting that *Dermacentor* species might play a role in the maintenance or transmission of the virus. However, the relatively low number of examined *Dermacentor* ticks (N=91) and the detection of TAMV RNA in only one pool represent important limitations that prevent any conclusion regarding vector competence. Previous surveys conducted in Türkiye have identified TAMV in *H. marginatum* collected from a gerbil (*Meriones tristrami*) and in *H. aegyptium* from a tortoise (*Testudo graeca*), indicating that multiple tick species may be involved in TAMV circulation [20-23]. Considering these findings, the potential role of *Dermacentor* and *Hyalomma* ticks, as well as their vertebrate hosts, in the maintenance and dissemination of TAMV across Türkiye and neighboring regions warrants further investigation. Future studies should aim to clarify the vector competence of *D. marginatus* and other sympatric tick species for TAMV through controlled experimental infections and broader field surveillance. Integrating entomological, serological, and molecular data from both animal and human populations would provide a more comprehensive understanding of TAMV ecology and its zoonotic potential in Türkiye and surrounding regions.

Detecting TAMV firstly in Osmaniye also suggests that TAMV has a widespread distribution out of previously stated areas in Türkiye. TAMV has recently been reported in *H. asiaticum* (Zhou et al., 2019; Moming et al., 2021), and a conducted seroprevalence study showed human TAMV infection among archived serum samples in the same region where the TAMV was detected in ticks in China (Moming et al., 2021). Another study conducted on ticks infesting dromedary camels (*Camelus dromedaries*) has demonstrated TAMV in *H. dromedarii* in Saudi Arabia (Zakham et al., 2021). Finally, TAMV is found in *H. asiaticum* and *D. nuttalli*, with serological evidence as different rates in domestic and wild rodents in China.

The SIMPLOT analysis and findings of the phylogenetic tree based on the partial L and full S segments of TAMV suggested that the OS44 was closely related to the TT1 isolate according to the Russian and Chinese strains. This could be explained by genetic diversity in the strains seen in Türkiye emerging through changes among themselves. An alternative explanation for this observation could be the absence of TAMV strains that promote genetic diversity in Türkiye to date. However, this situation can be clarified using more genetic data supported by field studies such as tick and domestic/wild animal surveys in Türkiye. In this study, the conserved motifs on the S segment, known to be responsible for the formation of the ribonucleoprotein complex and in vitro replication, were detected in the TAMV putative nucleocapsid proteins. Further studies are needed to determine the potential effects of the amino acid changes (explained in the result section) identified in both the OS44 and TT1 isolate on the functions of the NP. Considering the effectiveness of NP in treatment strategies against CCHFV, investigating the changes occurring in this region for TAMV may provide guidance for future studies aiming to follow similar approaches.

This study has several limitations that should be considered when interpreting the results.

(i) The relatively low minimum infection rate detected suggests that the sensitivity of the surveillance program may not have been sufficient to fully capture the prevalence of TAMV. Therefore, increasing the temporal and spatial resolution of surveillance efforts would be crucial for more accurate estimations of virus circulation. (ii) The limited number of *Dermacentor* ticks collected restricts the ability to evaluate this species as a competent vector for TAMV. Consequently, the potential role of *Dermacentor* in the virus transmission cycle remains inconclusive. Broader investigations, including a larger sample size and more diverse host and vector populations, are necessary to clarify the epidemiological role of this tick genus in TAMV ecology. (iii) Although genetic clustering analysis indicated internal diversity within the identified strains, the current dataset is not comprehensive enough to exclude potential introductions from outside the region. This underlines the need for more extensive genome-wide sequencing to better assess the phylogeographic patterns of TAMV and to identify possible routes of introduction or spread.

In conclusion, TBVs, such as CCHFV are often associated with severe clinical outcomes (Spengler et al., 2019). Consequently, the health risks posed by TAMV may be underestimated. In Türkiye, information on TAMV remains limited due to the lack of systematic surveillance programs and established diagnostic tools. Given that TAMV is considered a prototypical member of the Tamdy virus (*Orthonairovirus tomdiense*) species, its potential medical relevance to humans, as well as its distribution among ticks and animal hosts, warrants further comprehensive investigation. Finally, our findings not only revealed the genetic variants of TAMV circulating in the field but also offered perspective on changes in the NP gene, which may aid in identifying potential target regions for future diagnostic and therapeutic applications.
